# Shifts in the diversity of root endophytic microorganisms across the life cycle of the ratooning rice Jiafuzhan

**DOI:** 10.3389/fmicb.2023.1161263

**Published:** 2023-06-30

**Authors:** Meng Dong, Longqing Shi, Zhenxing Xie, Ling Lian, Junian Zhang, Zhaowei Jiang, Chunzhu Wu

**Affiliations:** Rice Research Institute, Fujian Academy of Agricultural Sciences, Fuzhou, China

**Keywords:** *16S rRNA*, *ITS*, rice root microbiota, endophytic microorganisms, *Bradyrhizobium*

## Abstract

The diversity of root endophytic microorganisms, which is closely related to plant life activities, is known to vary with the plant growth stage. This study on the ratooning rice Jiafuzhan explored the diversity of the root endophytic bacteria and fungi and their dynamics during the plant life cycle. By sequencing the 16S ribosomal ribonucleic acid (*16S rRNA*) and internal transcribed spacer (*ITS*) genes, 12,154 operational taxonomic units (OTUs) and 497 amplicon sequence variants (ASVs) were obtained, respectively. The root endophytic microorganisms of rice in the seedling, tillering, jointing, heading, and mature stages of the first crop and at 13, 25, and 60 days after regeneration (at the heading, full heading, and mature stages of the second crop, respectively) were analyzed using diversity and correlation analyses. There were significant differences in the α-diversity and β-diversity of root endophytic bacteria and fungi in the growth stage. Additionally, linear discriminant analysis (LDA) effect size (LEfSe) analysis revealed biomarker bacteria for each growth stage, but biomarker fungi did not exist in every stage. Moreover, the correlation analysis showed that the bacterial and fungal biomarkers interacted with each other. Furthermore, the nitrogen-fixing genus *Bradyrhizobium* existed in all growth stages. These findings indicate the pattern of root endophytic microorganisms of ratooning rice at different growth stages, and they provide new insights into the high yield of the second crop of ratooning rice (in light of the abundance of various bacteria and fungi).

## 1. Introduction

Root endophytic microorganisms promote plant growth, increase plant resistance to biotic and abiotic stresses *via* beneficial interactions with plants (Durán et al., 2018; Carrión et al., 2019), and facilitate nutrient absorption and utilization by plants (Zhang et al., 2019a). For example, 29–82% of nitrogen needed for maize growth can be provided by diazotrophic microbiota on the aerial roots of maize (Van Deynze et al., 2018). For *Arabidopsis thaliana*, the influence of phosphate starvation can be reduced by plant immunity by regulating the root endophytic microbiome (Tang et al., 2022). Moreover, hybrid maize can grow better with soil microorganisms (Wagner et al., 2021), and soil microbial diversity contributes to the super high yield of rice in Taoyuan (Yunnan, China). More diverse bacteria and fewer fungi, as well as nitrogen metabolism functions, may underlie the super high yield of rice (Zhong et al., 2020). Endophytes may contribute to the antioxidant activity in black rice, and the microbial community structure changes during the growth of the plant (Singha et al., 2021).

The structures of the root endophytic microbiomes of several plants including *Arabidopsis thaliana* (Chen et al., 2020a), rice (Edwards et al., 2015; Zhang et al., 2019a; Hu et al., 2020; Zhong et al., 2020), maize (Walters et al., 2018; Fadiji et al., 2020a,b), and sugarcane (Dong et al., 2018) have been analyzed using high-throughput sequencing. The soil characteristics in different regions affect microbial diversity (Zhang et al., 2019a; Kunda et al., 2021). The genotype of plants also has an impact on the diversity of root endophytic microorganisms, as reported in *Arabidopsis thaliana* (Bai et al., 2015), rice (Zhang et al., 2019a; Hu et al., 2020), maize (Walters et al., 2018), and sugarcane (Dong et al., 2018). The diversity of root endophytic microorganisms can even change with plant growth and plant development (Zhang et al., 2018). During the life cycle of rice, the relative abundance of Deltaproteobacteria increases, whereas the relative abundance of Betaproteobacteria, Firmicutes, and Gammaproteobacteria decreases (Zhang et al., 2018). Among the root endophytic diazotrophs of ratooning rice, *Bradyrhizobium* and *Azospirillum* are dominant genera throughout the growth and development stages (Dong et al., 2022).

Regenerated rice refers to the second crop of rice obtained from the tillers after harvesting, which does not necessitate using more land or resources. This is more beneficial to the environment than the cultivation of single-crop rice (Firouzi et al., 2018). The tiller number of ratooning rice depends on the use of chemical fertilizers, which alter the physicochemical properties of the soil. Biofertilizers have been applied to a variety of crops as an alternative to some chemical fertilizers and can be used as bioenhancers to improve plant growth and nutrient uptake (Rong et al., 2014; Zhang et al., 2019c). Plant growth-promoting bacteria isolated from the roots of ratooning rice play a significant role in promoting the growth of axillary buds in ratooning rice (Xu et al., 2020). However, the dynamic changes in the root endophytic bacteria and fungi of ratooning rice during its growth and development have not been reported in detail.

Jiafuzhan is an *indica* rice with strong ratooning ability (Cai, 2013). The growth period of the first crop is approximately 123 days; the spikelets differentiate 13 days after the first harvest, full heading occurs at approximately 25 days, and maturation occurs at approximately 60 days (Xie et al., 2019). Does the diversity of the root microbiome in the Full Heading and Mature stages of ratooning rice during the regenerated season resemble that of the Full Heading and Mature stages during the first season? The microbiome structure of ratooning rice still lacks systematic analysis. In this study, we used Jiafuzhan as the experimental material and conducted sampling at eight different growth stages. The changes in the root endophytic bacteria and fungi of the ratooning rice Jiafuzhan at different growth stages were studied by amplifying and sequencing the 16S ribosomal ribonucleic acid (*16S rRNA*) and internal transcribed spacer (*ITS*) genes. These results help to understand the changes in the root endophytic bacterial and fungal diversity in ratooning rice at different growth stages.

## 2. Materials and methods

### 2.1. Plants and cultivation

In mid-March 2019, seeds of the ratooning rice Jiafuzhan were sown on the farm (119.366E, 26.013N) of the Rice Research Institute, Fujian Academy of Agricultural Sciences in Fuzhou, China, and rice seedlings were transplanted by hand after 30 days. Samples were collected at the seedling, tillering, jointing, heading, and mature stages of the first crop and at 13, 25, and 60 days after regeneration (designated Regeneration 13 d, Regeneration 25 d, and Regeneration 60 d, respectively) at the heading, full heading, and mature stages of the second crop, respectively. There were three sample replicates per stage, giving a total of 24 samples. An area of 12 m^2^ per replicate and protective rows were set up. The rice stubble height was kept at 30 cm. Bud-promoting urea fertilizer (112.5 kg/hm^2^) was applied 18 days after the full heading of the first crop, seedling urea fertilizer (75 kg/hm^2^) was applied 3 days after harvesting, and panicle urea fertilizer (112.5 kg/hm^2^) was applied at the full heading stage of the second crop. The soil was watered on the same day to maintain a shallow-water layer, given the excessively dry soil after harvesting.

### 2.2. Sample collection

For the seedling stage, the roots were obtained 1 week after transplantation. For the other samples, the roots (10 cm underground) were obtained at the required growth stage. The samples were rinsed with running water to remove the surface soil, placed in a 50-ml tube containing 75% alcohol, underwent three rounds of shaking (at a frequency of 180 r/min) and cleaning, washed with sterile water 3–5 times, left in 20 g/L sodium hypochlorite solution for 10 min for disinfection, rinsed again with sterile water 3–5 times, dried using sterile filter paper, and stored at −80°C. The sterilized roots were placed on luria broth (LB) plates for 24 h to confirm that the disinfection was complete.

### 2.3. Library construction and sequencing of *16S rRNA* and *ITS* genes

Total genomic DNA was extracted from the root samples using an OMEGA Soil DNA Kit (M5635-02; Omega BioTek, Norcross, GA, USA), following the manufacturer's instructions. The DNA was, then, stored at −20°C before analysis. The quantity and quality of the extracted DNA were measured using a NanoDrop NC2000 spectrophotometer (Thermo Fisher Scientific, Waltham, MA, USA) and agarose gel electrophoresis, respectively. Polymerase chain reaction (PCR) amplification using primers for 16S rRNA (338F: ACTCCTACGGGAGGCAGCA, 806R: GGACTACHVGGGTWTCTAAT, targeting the V3-V4 region) (Caporaso et al., 2011) and ITS (ITS-F: GGAAGTAAAAGTCGTAACAAGG, ITS-R: GCTGCGTTCTTCATCGATGC) (Zhang et al., 2019b) was carried out with negative control (no template added). The PCR involved 5 μl of buffer (5 × ), 0.25 μl (5 U/μl) of FastPfu DNA Polymerase (TransGen Biotech), 2 μl (2.5 mM) of dNTPs, 1 μl (10 μM) of each of forward and reverse primers, 1 μl of DNA template, and 14.75 μl of ddH_2_O. Thermal cycling consisted of initial denaturation at 98°C for 5 min, followed by 25 cycles of denaturation at 98°C for 30 s, annealing at 53°C for 30 s, and extension at 72°C for 45 s, with a final extension of 5 min at 72°C. The amplified products were purified and recovered using Vazyme VAHTS DNA Clean Beads (Vazyme, Nanjing, China). Then, they underwent fluorescence-based quantification using Quant-iT PicoGreen dsDNA Assay Kit (Invitrogen, Carlsbad, CA, USA) and an FLx800 microplate reader (BioTek). Based on the quantification results and the sequencing volumes required for samples at each stage, the samples were mixed in appropriate proportions. A sequencing library was constructed using a TruSeq Nano DNA LT Library Prep Kit (Illumina) and quantified using a Quant-iT PicoGreen dsDNA Assay Kit on a QuantiFluor dsDNA System (Promega Corporation). Thereafter, amplicons were pooled in equal amounts, and paired-end 2 × 250 bp sequencing was performed on an Illumina MiSeq platform with MiSeq Reagent Kit v3 at Shanghai Personal Biotechnology Co., Ltd. (Shanghai, China).

### 2.4. Bioinformatics analyses

Microbiome bioinformatics analyses were performed using QIIME2 2019.4 (Bolyen et al., 2018), according to the official tutorials (https://docs.qiime2.org/2019.4/tutorials/), with slight modifications. In brief, raw sequence data were demultiplexed using the demux plugin and underwent primer removal using the cutadapt plugin (Martin, 2011). Regarding the bacterial sequences, the sequences were then merged, quality filtered, and dereplicated using the Vsearch plugin. The unique sequences were then clustered, chimera removal was conducted (using uchime denovo), and the non-chimera sequences were reclustered to generate representative operational taxonomic unit (OTU) sequences and an OTU table. Non-singleton amplicon sequence variants (ASVs) were aligned using mafft (Katoh et al., 2002) and used to construct a phylogeny using fasttree 2 (Price et al., 2009). Regarding the fungal sequences, the sequences were quality filtered, denoised, merged, and underwent chimera removal using the DADA2 plugin (Callahan et al., 2016). Taxonomy was assigned to the ASVs using the classify-sklearn naïve Bayes taxonomy classifier in the feature-classifier plugin (Bokulich et al., 2018) against the UNITE 8.0 database (Kõljalg et al., 2013).

The sequencing data were analyzed statistically using R 3.5.1. After the samples were rarefied, α-diversity and β-diversity analyses were conducted using the diversity plugin. OTU-level α-diversity indices (Observed species, Simpson index, Pielou's evenness, and Good's coverage) were calculated using the OTU/ASV tables in QIIME2, and the results were visualized using boxplots. β-diversity analysis was performed to investigate the structural variation of microbial communities among samples using Jaccard metrics, visualized using principal coordinate analysis (PCoA), non-metric multidimensional scaling (NMDS), and unweighted pair-group method with arithmetic mean (UPGMA) hierarchical clustering (Ramette, 2007). The significance of the differentiation of microbial communities among groups was assessed by permutational multivariate analysis of variance (PERMANOVA) (McArdle and Anderson, 2001), analysis of similarities (ANOSIM) (Clarke, 1993; Warton et al., 2012), Adonis (multivariate analysis of variance) (Anderson, 2001), and PERMDISP (Anderson et al., 2006) using QIIME2.

The numbers of shared and unique endophytic bacterial/fungal OTUs at the various growth stages were visualized using Venn diagrams. Linear discriminant analysis (LDA) effect size (LEfSe) was performed to detect bacterial/fungal biomarkers for the various growth stages (Segata et al., 2011). Spearman's correlation analysis was used to determine the relationships between the root endophytic bacteria and fungi (*p* < 0.05 indicated statistical significance) using OmicShare tools, a free online platform for data analysis (https://www.omicshare.com/tools).

## 3. Results

### 3.1. Sample collection, sequencing, and analysis

**Figure 1:**
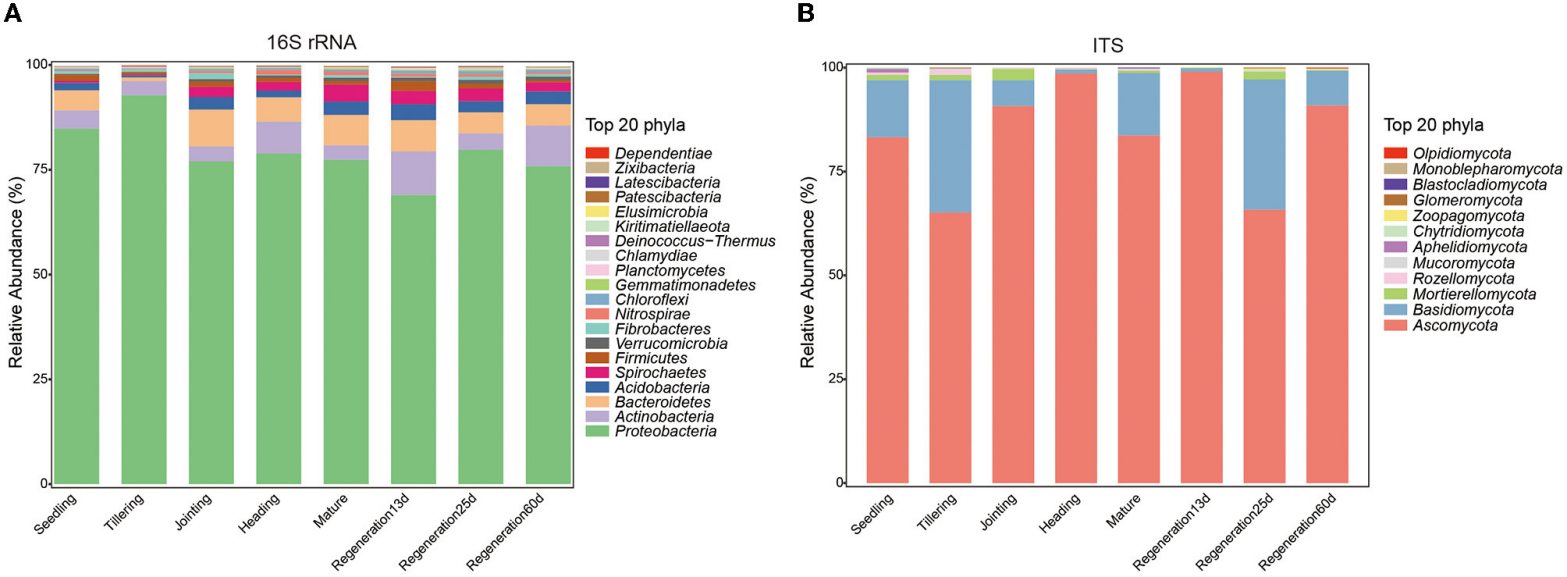
Differences in root endophytic **(A)** bacteria and **(B)** fungi at different growth stages of ratooning rice.

To explore the changes in the root endophytic microbial communities during the growth of Jiafuzhan ratooning rice in the field, 24 samples at 8 growth stages were collected from the farm of the Rice Research Institute in Fuzhou, China. Overall, 2,649,377 high-quality bacterial sequences (mean: 110,391 per sample; range: 71,974–140,477) and 2,821,669 high-quality fungal sequences (mean: 117,570 per sample; range: 93,091–145,412) were obtained (Supplementary Table S1), resulting in 12,154 OTUs and 497 ASVs. The number of bacterial OTUs was the lowest at the tillering stage, while the number of fungal OTUs was the lowest at the jointing and heading stages (Supplementary Figure S1). Overall, the most dominant bacterial phyla (across all samples) were Proteobacteria (79.45%), Actinobacteria (5.81%), Bacteroidetes (5.64%), Acidobacteria (2.44%), and Spirochaetes (2.23%), while the most dominant fungal phyla were Ascomycota (84.65%), Basidiomycota (13.56%), Mortierellomycota (1.01%), Rozellpmycota (0.30%), and Mucoromycota (0.12%) (Figure 1). The abundance of these phyla changed among the growth stages (e.g., Proteobacteria peaked in the tillering stage and was the lowest at Regeneration 13 d, and Ascomycota peaked at Regeneration 13 d and was the lowest at the tillering stage).

### 3.2. Influences of growth stage on α-diversity

**Figure 2:**
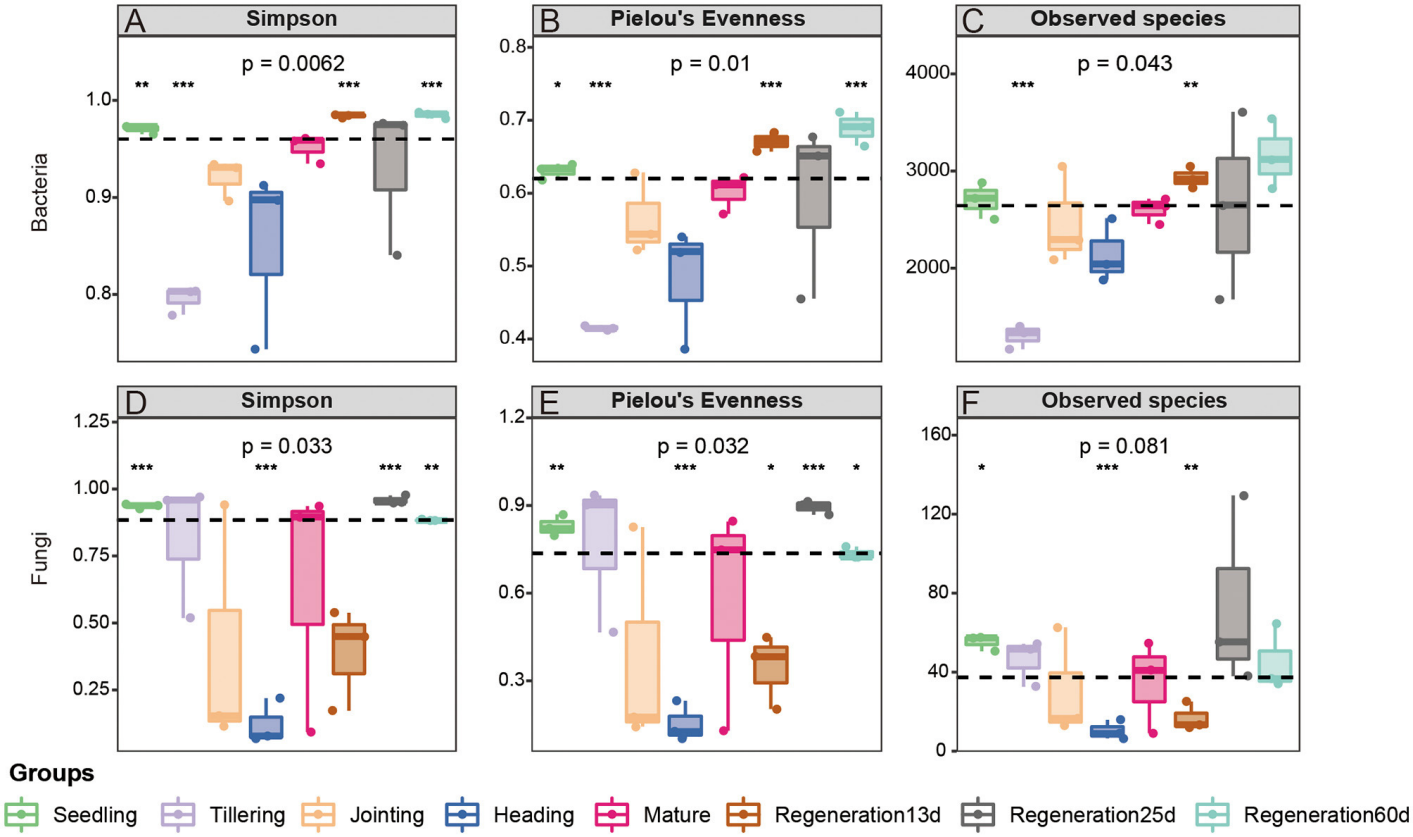
α-diversity of root **(A–C)** bacteria and **(D–F)** fungi at different growth stages of ratooning rice.

The Simpson index, Pielou's Evenness, Observed species, and Good's coverage were used to compare the diversity, evenness, number of species, and coverage rate, respectively, of the root endophytic bacteria and fungi at various growth stages. Regarding bacteria, Regeneration 60 d had the highest diversity (Simpson index: 0.985208667) and the largest number of species (Observed species: 3161.51), while the tillering stage had the lowest diversity (Simpson index: 0.795443) and the smallest number of species (Observed species: 1301.8). The α-diversity of root bacteria at maturity in the rice regeneration season (Regeneration 60 d) was higher than that in the first season (mature stage), while the α-diversity of root endophytic fungi was similar in these two stages. The α-diversity of root endophytic fungi at the different stages decreased as follows: the heading stage, Regeneration 13 d, jointing stage, mature stage, tillering stage, Regeneration 60 d, seedling stage, and Regeneration 25 d (Figure 2).

### 3.3. Influences of growth stage on β-diversity

**Figure 3:**
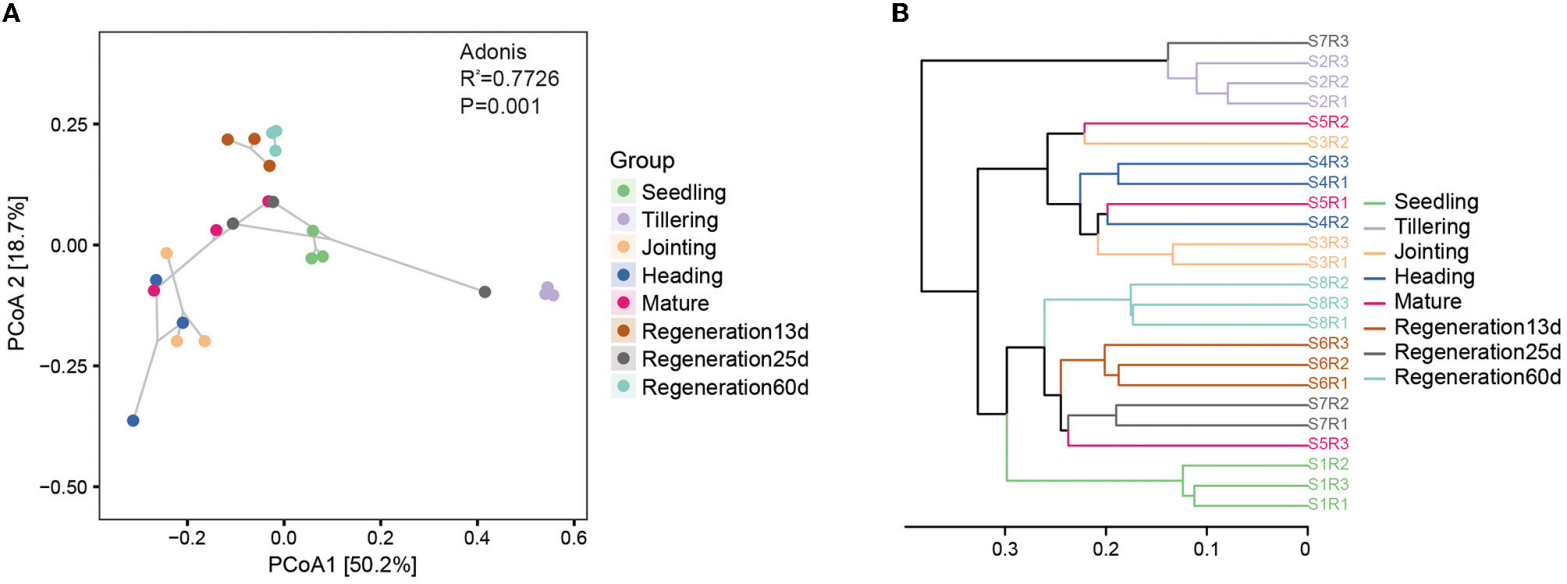
β-diversity of root endophytic bacteria at different growth stages of ratooning rice. **(A)** PCoA and **(B)** UPGMA cluster analysis. Seedling stage samples: S1R1, S1R2, and S1R3; tillering stage samples: S2R1, S2R2, and S2R3; jointing stage samples: S3R1, S3R2, and S3R3; heading stage samples: S4R1, S4R2, and S4R3; mature stage samples: S5R1, S5R2, and S5R3; Regeneration13d samples: S6R1, S6R2, and S6R3; Regeneration25d samples: S7R1, S7R2, and S7R3; Regeneration60d samples: S8R1, S8R2, and S8R3.

The β-diversity of the root endophytic bacteria and fungi was analyzed using weighted and unweighted UniFrac distances. Regarding the bacteria, the Adonis test based on the weighted UniFrac distance was significant (*R*^2^ = 0.7726, *P* = 0.001). PCoA based on the weighted UniFrac distance also showed that the bacterial diversity varied by the growth stage. Specifically, there were large differences among the seedling, tillering, and jointing stages, while the differences among the jointing, heading, and mature stages were smaller, and Regeneration 13 d and Regeneration 60 d differed a lot from the other stages (Figure 3A). The PCoA and the Jaccard distance-based UPGMA cluster analysis concurred with each other. Specifically, the UPGMA cluster analysis showed that the jointing, heading, and mature stages clustered together, as did the regeneration stages, while the tillering stage did not cluster with any other stages, nor did the seedling stage (Figure 3B).

Regarding the fungi, the Adonis test was significant (*R*^2^ = 0.4656, *P* = 0.002). However, for the fungi, PCoA and the UPGMA cluster analysis gave slightly different results. According to PCoA, Regeneration 13 d was different from other growth stages, while the tillering, jointing, heading, and mature stages differed slightly (Supplementary Figure S2A). According to the UPGMA cluster analysis, the seedling stage, Regeneration 13 d, and Regeneration 60 d clustered together, and the tillering, jointing, and heading stages clustered together (Supplementary Figure S2B).

### 3.4. Influences of growth stage on root endophytic bacteria and fungi

**Figure 4:**
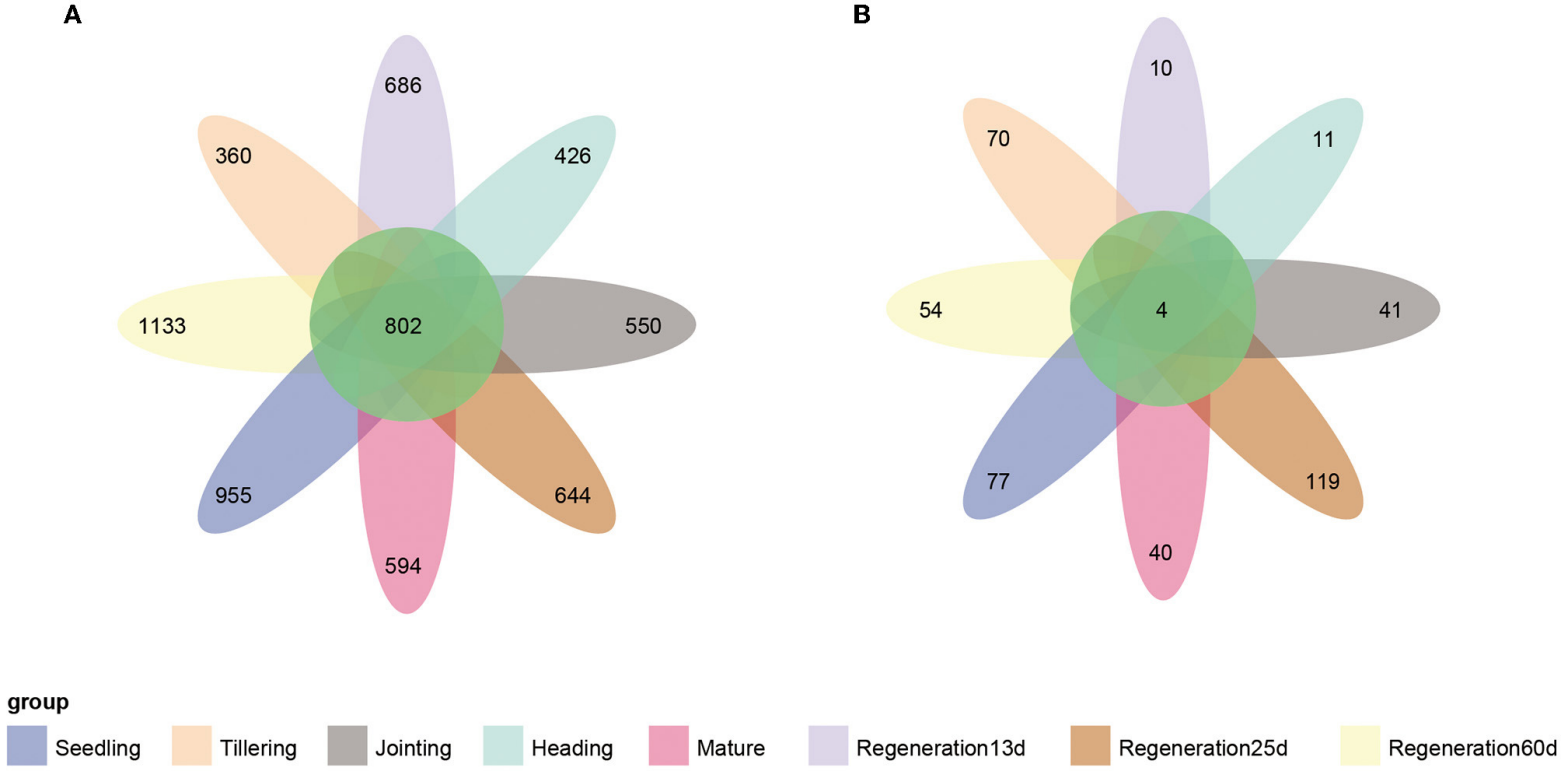
Venn diagrams of the number of endophytic **(A)** bacterial and **(B)** fungal OTUs at each growth stage of ratooning rice.

The Venn diagram showed that there were 802 core bacterial OTUs shared by all eight growth stages (Figure 4A). At the phylum level, Proteobacteria (60.7%) had the highest relative abundance, followed by Actinobacteria (13.5%) (Supplementary Figure S3A). At the genus level, *Anaeromyxobacter* had the highest relative abundance, followed by *Halomonas, Geobacter, Sideroxydans*, and *Bradyrhizobium* (Supplementary Figure S3B). Regarding the root endophytic fungi, there were only four core ASVs shared by all 8 growth stages (Figure 4B), and these belonged to the genera *Alternaria* (phylum Ascomycota) and *Chaetomium* (phylum Basidiomycota) (Supplementary Figures S3C, D).

To further compare the bacterial composition at each growth stage, a heatmap of the mean abundance of the bacterial genera was constructed, which showed that the enriched root endophytic bacteria varied by the growth stage (Supplementary Figure S4). The seedling, tillering, and jointing stages were dominated by four (*Pseudomonas, Hydrogenophaga, Flavobacterium, and Uniginosibacterium*), two (*Halomonas and Cupriavidus*), and five (*Sulfuritalea, Candidatus, Koribacter, Roseimarinus*, and *Treponema*) genera, respectively. The genera *Actinobacteria bacterium* CG2_30_50_142 and *Streptomyces* predominantly existed at the heading stage. *Geobacter* and *Bradyrhizobium* accounted for a large proportion at the mature stage. At Regeneration 13 d, Regeneration 25 d, and Regeneration 60 d, there were seven (*Dechloromonas, Anaerobacterium, Treponema, Desulfovibrio, Pleomorphomonas, Rhizomicrobium*, and *Dongia*), two (*Leptonema* and *Ellin6067*), and 10 (*Novosphingobium, Afipia, Acidibacter, Dokdonella, Sphingobium, Haliangium, Ancalomicrobium*, Subgroup 10, *Thiobacillus*, and *Devosia*) genera, respectively.

**Figure 5:**
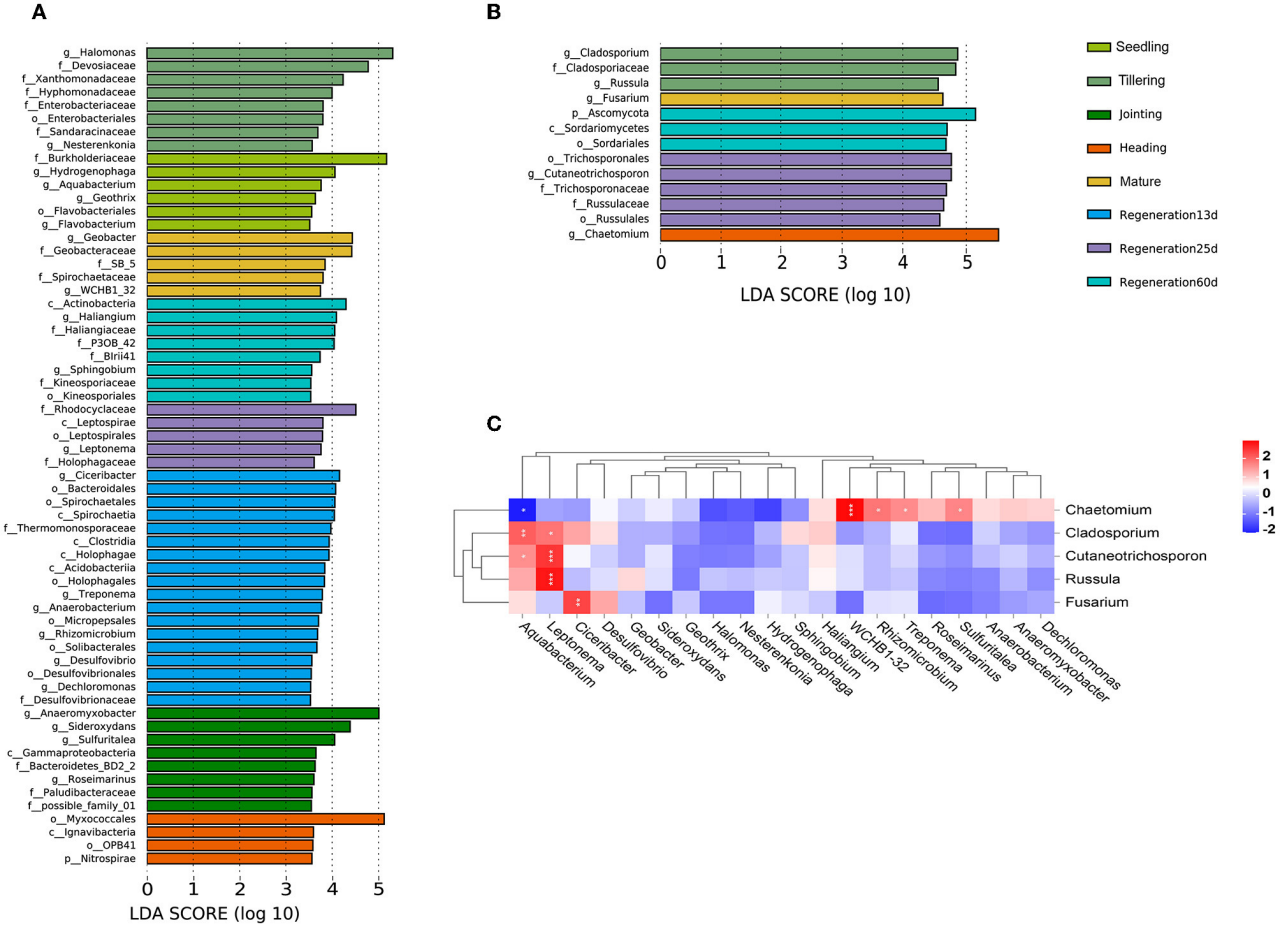
Associations among root endophytic bacteria and fungi of ratooning rice. LDA histograms of significant (LDA(log_10_) >3.5) **(A)** bacterial and **(B)** fungal species. **(C)** Heatmap of Spearman's correlations among bacteria and fungi. *, **, and *** denote significance at 0.05, 0.01, and 0.001 levels, respectively.

LEfSe analysis was performed to identify bacterial and fungal groups with significant differences at different growth stages. Based on an LDA(log_10_) > 2.0 and *P* < 0.05, there were 283 and 13 distinct bacterial and fungal groups, respectively, among the different growth stages (Supplementary Tables S2, S3). Based on LDA(log_10_) > 3.5 and *P* < 0.05, there were 62 and 13 distinct bacterial and fungal groups, respectively (Figures 5A, B). Based on LDA(log_10_) > 4.0 and *P* < 0.05, there were two bacterial biomarkers (f_Burkholderiaceae, g_Hydrogenophaga) in the seedling group, three (g_Halomonas, f_Devosiaceae, and f_Xanthomonadaceae) in the tillering group, three (g_Anaeromyxobacter, g_Sideroxydans, and g_Sulfuritalea) in the jointing group, one (o_Myxococcales) in the heading group, two (g_Geobacter, f_Geobacteraceae) in the mature group, four (g_Ciceribacter, o_Bacteroidales, o_Spirochaetales, and c_Spirochaetia) in the Regeneration 13 d group, one (f_Rhodocyclaceae) in the Regeneration 25 d group, and four (c_Actinobacteria, g_Haliangium, f_Haliangiaceae, and f_P3OB_42) in the Regeneration 60 d group. Additionally, there were three fungal biomarkers (g_Cladosporium, f_Cladosporiaceae, and g_Russula) in the tillering group, one (g_Chaetomium) in the heading group, one (g_Fusarium) in the mature group, five (o_Trichosporonales, g_Cutaneotrichosporon, f_Trichosporonaceae, f_Russulaceae, and o_Russulales) in the Regeneration 25 d group, and three (p_Ascomycota, c_Sordariomycetes, and o_Sordariales) in the Regeneration 60 d group. Interestingly, there were no fungi with LDA(log_10_) >3.5 in the seedling, jointing, or Regeneration 13 d groups.

A heatmap of the Spearman correlation coefficients was used to visualize the associations between the bacteria and fungi identified in the LEfSe analysis (Figure 5C). Only Chaetomium had a significant negative correlation with Aquabacterium (0.01 < *p* < 0.05). Chaetomium was also significantly positively correlated with WCHB1-32 (*p* ≤ 0.001), Rhizomicrobium, Treponema, and Sulfuritalea (0.01 < *p* < 0.05). Cutaneotrichosporon and Russula were significantly positively correlated with Leptonema (*p* ≤ 0.001). Fusarium was significantly positively correlated with Ciceribacter (0.01 < *p* < 0.01).

## 4. Discussion

Endophytic microorganisms in plant roots impact host plant health by influencing plant growth, development, resistance to biotic/abiotic stresses, and nutrient absorption and utilization (Müller et al., 2016; Toju et al., 2018; Mehlferber et al., 2022; Qi et al., 2022; Shekhawat et al., 2022; Tang et al., 2022). Studies have been carried out on the root endophytic microorganisms of rice at different stages (Edwards et al., 2017; Zhang et al., 2018), but there are few reports on ratooning rice. Our results revealed that under field conditions, the enriched bacterial phyla included Actinobacteria, Bacteroidetes, and Acidobacteria (Figure 1A), which is consistent with the root endophytic bacteria that were previously found to be enriched in Nipponbare and IR24 (Hu et al., 2020). The most dominant fungal phyla were Ascomycota, Basidiomycota, Mortierellomycota, Rozellpmycota, and Mucoromycota (Figure 1B), and the most dominant genus, *Chaetomium* (phylum Ascomycota) (Supplementary Figure S3D), promotes maize growth by regulating the expression of hormone-related genes in roots (Li et al., 2022) and produces microbial nanoparticles that can protect rice against rice blast fungus (Song et al., 2020).

Our α-diversity analysis revealed that the growth stage influenced the α-diversity of root endophytic bacteria and fungi (Figure 2), as indicated in previous studies (Zhang et al., 2018; Hu et al., 2020). The growth and development of plants are linked to the root endophytic microorganisms, and research on the root endophytic microorganisms at different growth stages of single-crop rice has shown that microorganism diversity is highest at the heading stage (Edwards et al., 2018; Zhang et al., 2018). In this study, the fungal diversity was the highest at the heading stage, followed by Regeneration 13 d and the jointing stage, and it was the lowest at the seedling stage followed by Regeneration 25 d. However, the bacterial diversity was the highest at Regeneration 60 d, followed by Regeneration 13 d and the seedling stage, and it was the lowest at the heading stage followed by the tillering stage (Figure 2). The difference in bacterial diversity between this study and previous studies may be caused by the different cultivation methods between ratooning rice (first crop fertilization) and single-crop rice (Liu et al., 2019; Chen et al., 2020b), after 18 days of full heading in the first season and 3 days of harvest of ratooning rice, bud promoting fertilizer would be applied, irrigation would be carried out after harvest of the first season to ensure the growth of ratoon buds, these may also be the main factors affecting the diversity of endophytic bacteria between the regenerating season and the first season in ratooning rice.

Our β-diversity analysis of root endophytic bacteria and fungi showed that there were remarkable differences among growth stages. Regarding bacteria, the regeneration stages clustered together, and the seedling stage did not cluster with the other stages. Furthermore, the root endophytic microorganisms of the first crop changed over time so that the microorganisms at the end stages of the first crop greatly differed from the microorganisms at the early stage (Figure 3; Supplementary Figure S2). Based on the results, the changes in the root endophytic microorganisms of ratooning rice after the first harvest in our study may be due to the rebuilt bacterial communities in the new roots after the first harvest (Zhang et al., 2018) and applying fertilizers before and after the first harvest of regenerated rice (Chen et al., 2022).

Biological nitrogen fixation can provide nitrogen for plants, and many endophytic nitrogen-fixing bacteria have been found in rice roots (Ji et al., 2014; Dong et al., 2023). Nitrogen-fixing *Bradyrhizobium* can improve the growth and yield of rice (Padukkage et al., 2021). We found that nitrogen-fixing bacteria were enriched at various growth stages, such as *Bradyrhizobium* and *Allorhizobium*-*Neorhizobium*-*Pararhizobium*-*Rhizobium* (Supplementary Figure S4). *Bradyrhizobium* has also previously been found to be a dominant genus at all growth stages of ratooning rice (Dong et al., 2022). However, further research is needed to validate the contribution of nitrogen-fixing bacteria to nitrogen absorption in rice.

As rice grows and develops, root endophytic microorganisms change, which is closely related to the host plant and the environment (Edwards et al., 2018; Zhang et al., 2018). Nevertheless, there are few studies on the changes in the diversity of rice root endophytic fungi. Our results show that both the relative abundance and diversity were lower for fungi than bacteria (Figures 1, 4). This was consistent with previous research showing that the high yield of rice is related to root endophytic microorganisms; the richer the bacterial population, the lower the fungal population, which increases the rice yield (Zhong et al., 2020). The improvement of mycorrhizal characteristics by fungi-correlated bacteria was first verified in a study of mycorrhiza helper bacteria (MHB) (Garbaye, 1994). Both the genera *Pseudomonas* (phylum Proteobacteria) and *Bacillus* (phylum Firmicutes) are known to interact with arbuscular mycorrhizal fungi (Scheublin et al., 2010; Turrini et al., 2018). In this study, most of the correlated bacterial and fungal biomarkers (for the various growth stages) were positively correlated rather than negatively correlated (*p* < 0.05). *Chaetomium* was a fungal biomarker for the heading stage; it is related to rice blast resistance and is a plant growth-promoting fungus (PGPF). The bacteria that were positively correlated (*p* < 0.05) (Figure 5) with *Chaetomium* (Rhizomicrobium, WCHB1_32, Treponema, and Sulfuritalea) are plant growth-promoting bacteria (PGPB) (Tann and Soytong, 2016; Jiao and Soytong, 2018). The presence of numerous bacteria that were positively correlated with *Chaetomium* may enhance the disease resistance of ratooning rice. The yield of regenerated rice depends on the germination of regenerated axillary buds, and the growth-promoting strains isolated from the rhizosphere can effectively promote the growth of regenerated rice axillary buds (Xu et al., 2020). During the mature stages of the first season, nitrogen-fixing bacteria become enriched (Supplementary Figure S4), supplying essential nutrients for the germination of regenerated axillary buds, which, in turn, influences the overall yield. Previous research showed that increased bacterial diversity and decreased fungal diversity increase rice yields (Zhong et al., 2020), and increased bacterial diversity may also contribute to Jiafuzhan's high second crop yield.

In conclusion, this study revealed for the first time the diversity of endophytic microorganisms in ratooning rice roots. The bacterial diversity in roots was higher than the fungal diversity, and both were related to the growth stage. Many bacterial genera (such as *Anaeromyxobacter, Halomonas*, and *Bradyrhizobium*) dominated in all growth stages. However, how ratooning rice growth and development stages influence the changes in the root endophytic bacteria and fungi and whether and how interactions exist among root endophytic bacteria and fungi remain unknown. In the future, in-depth research will be carried out on the interactions between root endophytic bacteria, root endophytic fungi, and host plants.

## Data availability statement

The datasets presented in this study can be found in online repositories. The names of the repository/repositories and accession number(s) can be found at: https://www.ncbi.nlm.nih.gov/, PRJNA87706; https://www.ncbi.nlm.nih.gov/, PRJNA877777.

## Author contributions

MD, ZJ, and CW designed the research. MD, LS, ZX, LL, and JZ prepared the materials. MD, LL, and JZ analyzed the data. MD and LS wrote the draft manuscript. All authors contributed to the article and approved the submitted version.

## Supplementary material

Supplementary Figure S1 OTU taxonomic statistics of root endophytic **(A)** bacteria and **(B)** fungi. Seedling stage samples: S1R1, S1R2, and S1R3; tillering stage samples: S2R1, S2R2, and S2R3; jointing stage samples: S3R1, S3R2, and S3R3; heading stage samples: S4R1, S4R2, and S4R3; mature stage samples: S5R1, S5R2, and S5R3; Regeneration13d samples: S6R1, S6R2, and S6R3; Regeneration25d samples: S7R1, S7R2, and S7R3; Regeneration60d samples: S8R1, S8R2, and S8R3.

Supplementary Figure S2 β-diversity of root endophytic fungi at different growth stages of ratooning rice. **(A)** PCoA and **(B)** UPGMA cluster analysis. Seedling stage samples: S1R1, S1R2, and S1R3; tillering stage samples: S2R1, S2R2, and S2R3; jointing stage samples: S3R1, S3R2, and S3R3; heading stage samples: S4R1, S4R2, and S4R3; mature stage samples: S5R1, S5R2, and S5R3; Regeneration13d samples: S6R1, S6R2, and S6R3; Regeneration25d samples: S7R1, S7R2, and S7R3; Regeneration60d samples: S8R1, S8R2, and S8R3.

Supplementary Figure S3 OTU occurrence frequency of root endophytic bacterial **(A)** phyla and **(B)** genera and fungal **(C)** phyla and **(D)** genera at different growth stages of ratooning rice.

Supplementary Figure S4 Heatmap of the mean abundance of the top 50 root endophytic bacteria at different growth stages of ratooning rice.

Supplementary Table S1 Sequencing statistics for different samples. Seedling stage samples: S1R1, S1R2, and S1R3; tillering stage samples: S2R1, S2R2, and S2R3; jointing stage samples: S3R1, S3R2, and S3R3; heading stage samples: S4R1, S4R2, and S4R3; mature stage samples: S5R1, S5R2, and S5R3; Regeneration13d samples: S6R1, S6R2, and S6R3; Regeneration25d samples: S7R1, S7R2, and S7R3; Regeneration60d samples: S8R1, S8R2, and S8R3.

Supplementary Table S2 Top 50 root endophytic bacteria of ratooning rice (based on mean abundance).

Supplementary Table S3 Top 50 root endophytic fungi of ratooning rice (based on mean abundance).

## References

[B1] Anderson M. J. (2001). A new method for non-parametric multivariate analysis of variance. Austral Ecol. 26, 32–46. 10.1111/j.1442-9993.2001.01070.pp.x

[B2] Anderson M. J. Ellingsen K. E. McArdle B. H. (2006). Multivariate dispersion as a measure of beta diversity. Ecol. Lett. 9, 683–693. 10.1111/j.1461-0248.2006.00926.x 16706913

[B3] Bai Y. Müller D. B. Srinivas G. Garrido-Oter R. Potthoff E. Rott M.. (2015). Functional overlap of the Arabidopsis leaf and root microbiota. Nature. 528, 364–369. 10.1038/nature16192 26633631

[B4] Bokulich N. A. Kaehler B. D. Rideout J. R. Dillon M. Bolyen E. Knight R.. (2018). Optimizing taxonomic classification of marker-gene amplicon sequences with QIIME 2′s q2-feature-classifier plugin. Microbiome. 6, 90. 10.1186/s40168-018-0470-z 29773078 PMC5956843

[B5] Bolyen E. Rideout J. R. Dillon M. R. Bokulich N. A. Abnet C. Al-Ghalith G. A.. (2018). QIIME 2: Reproducible, interactive, scalable, and extensible microbiome data science. PeerJ Preprints. 6, e27295ve27292. 10.7287/peerj.preprints.27295v2 31341288 PMC7015180

[B6] Cai G. (2013). Screening of regenerative capacity of ratoon high-quality rice under low stubble condition. Fujian Agric. Sci. Technol. 1–4. 10.13651/j.cnki.fjnykj.2013.08.0236404524

[B7] Callahan B. J. McMurdie P. J. Rosen M. J. Han A. W. Johnson A. J. Holmes S. P. (2016). DADA2: high-resolution sample inference from Illumina amplicon data. Nat. Methods. 13, 581–583. 10.1038/nmeth.3869 27214047 PMC4927377

[B8] Caporaso J. G. Lauber C. L. Walters W. A. Berg-Lyons D. Lozupone C. A. Turnbaugh P. J.. (2011). Global patterns of 16S rRNA diversity at a depth of millions of sequences per sample. Proc. Natl. Acad. Sci. U S A. 108(Suppl 1), 4516–4522. 10.1073/pnas.1000080107 20534432 PMC3063599

[B9] Carrión V. J. Perez-Jaramillo J. Cordovez V. Tracanna V. de Hollander M. Ruiz-Buck D.. (2019). Pathogen-induced activation of disease-suppressive functions in the endophytic root microbiome. Science. 366, 606–612. 10.1126/science.aaw9285 31672892

[B10] Chen J. Sharifi R. Khan M. S. S. Islam F. Bhat J. A. Kui L.. (2022). Wheat microbiome: structure, dynamics, and role in improving performance under stress environments. Front. Microbiol. 12, 821546. 10.3389/fmicb.2021.821546 35095825 PMC8793483

[B11] Chen T. Nomura K. Wang X. Sohrabi R. Xu J. Yao L.. (2020a). A plant genetic network for preventing dysbiosis in the phyllosphere. Nature. 580, 653–657. 10.1038/s41586-020-2185-0 32350464 PMC7197412

[B12] Chen X. Xia Y. Rui Y. Ning Z. Hu Y. Tang H.. (2020b). Microbial carbon use efficiency, biomass turnover, and necromass accumulation in paddy soil depending on fertilization. Agric. Ecosyst. Environ. 292, 106816. 10.1016/j.agee.2020.106816

[B13] Clarke K. R. (1993). Non-parametric multivariate analyses of changes in community structure. Austral. J. Ecol. 18, 117–143. 10.1111/j.1442-9993.1993.tb00438.x

[B14] Dong M. Shi L. Xie Z. Lian L. Wu C. Zhang J.. (2023). Isolation, identification and growth promotion of endophytic nitrogen fixing bacteria from rice roots. J. Northwest A&F Univ. 51, 31–39. 10.13207/j.cnki.jnwafu.2023.01.005

[B15] Dong M. Xie Z. Shi L. Lian L. Zhang J. Jiang Z.. (2022). Diversity analysis of endophytic nitrogen fixing bacteria in roots for ratooning rice. Fujian Sci. Technol. Rice Wheat. 40, 13–18.

[B16] Dong M. Yang Z. Cheng G. Peng L. Xu Q. Xu J. (2018). Diversity of the bacterial microbiome in the roots of four saccharum species: S. spontaneum, S. robustum, S. barberi, and S. officinarum. Front. Microbiol. 9, 267. 10.3389/fmicb.2018.00267 29515548 PMC5826347

[B17] Durán P. Thiergart T. Garrido-Oter R. Agler M. Kemen E. Schulze-Lefert P.. (2018). Microbial interkingdom interactions in roots promote arabidopsis survival. Cell. 175, 973–983.e14. 10.1016/j.cell.2018.10.020 30388454 PMC6218654

[B18] Edwards J. Johnson C. Santos-Medellín C. Lurie E. Podishetty N. K. Bhatnagar S.. (2015). Structure, variation, and assembly of the root-associated microbiomes of rice. Proc. Natl. Acad. Sci. U S A. 112, E911–920. 10.1073/pnas.1414592112 25605935 PMC4345613

[B19] Edwards J. Santos-Medellín C. Liechty Z. Nguyen B. Lurie E. Eason S.. (2017). Compositional shifts in the root microbiota track the life-cycle of field-grown rice plants. bioRxiv 166025. 10.1101/166025 PMC5841827 29474469

[B20] Edwards J. A. Santos-Medellín C. M. Liechty Z. S. Nguyen B. Lurie E. Eason S.. (2018). Compositional shifts in root-associated bacterial and archaeal microbiota track the plant life cycle in field-grown rice. PLoS Biol. 16, e2003862. 10.1371/journal.pbio.2003862 29474469 PMC5841827

[B21] Fadiji A. E. Ayangbenro A. S. Babalola O. O. (2020a). Metagenomic profiling of the community structure, diversity, and nutrient pathways of bacterial endophytes in maize plant. Antonie Van Leeuwenhoek. 113, 1559–1571. 10.1007/s10482-020-01463-w 32803452

[B22] Fadiji A. E. Ayangbenro A. S. Babalola O. O. (2020b). Organic farming enhances the diversity and community structure of endophytic archaea and fungi in maize plant: a shotgun approach. J. Soil Sci. Plant Nutr. 20, 2587–2599. 10.1007/s42729-020-00324-9

[B23] Firouzi S. Nikkhah A. Aminpanah H. (2018). Rice single cropping or ratooning agro-system: which one is more environment-friendly? Environ. Sci. Pollut. Res. Int. 25, 32246–32256. 10.1007/s11356-018-3076-x 30225691

[B24] Garbaye J. (1994). Tansley review no. 76 helper bacteria: a new dimension to the mycorrhizal symbiosis. New Phytol. 128, 197–210. 10.1111/j.1469-8137.1994.tb04003.x 33874371

[B25] Hu Y. Dai R. Liu Y. Zhang J. Hu B. Chu C.. (2020). Analysis of rice root bacterial microbiota of Nipponbare and IR24. Hereditas. 42, 506–518. 10.16288/j.yczz.20-070 32431301

[B26] Ji S. H. Gururani M. A. Chun S. C. (2014). Isolation and characterization of plant growth promoting endophytic diazotrophic bacteria from Korean rice cultivars. Microbiol. Res. 169, 83–98. 10.1016/j.micres.2013.06.003 23871145

[B27] Jiao J. S. Soytong K. (2018). Nano-particles from chaetomium against rice blast. Cold Spring Harbor Laboratory. bioRxiv. 10.1101/339283

[B28] Katoh K. Misawa K. Kuma K. Miyata T. (2002). MAFFT: a novel method for rapid multiple sequence alignment based on fast Fourier transform. Nucleic Acids Res. 30, 3059–3066. 10.1093/nar/gkf436 12136088 PMC135756

[B29] Kõljalg U. Nilsson R. H. Abarenkov K. Tedersoo L. Taylor A. F. Bahram M.. (2013). Towards a unified paradigm for sequence-based identification of fungi. Mol. Ecol. 22, 5271–5277. 10.1111/mec.12481 24112409

[B30] Kunda P. Mukherjee A. Dhal P. K. (2021). Insights into endophytic bacterial diversity of rice grown across the different agro-ecological regions of West Bengal, India. World J. Microbiol. Biotechnol. 37, 184. 10.1007/s11274-021-03153-9 34580777

[B31] Li Y. Hu D. Tan J. Mei H. Wang Y. Li H.. (2022). Chaetomium uniseriatum promotes maize growth by accelerating straw degradation and regulating the expression of hormone responsive genes. Chinese Bulletin of Botany. 57, 422–433. 10.11983/CBB21147

[B32] Liu Y. Ge T. Ye J. Liu S. Shibistova O. Wang P.. (2019). Initial utilization of rhizodeposits with rice growth in paddy soils: Rhizosphere and N fertilization effects. Geoderma. 338, 30–39. 10.1016/j.geoderma.2018.11.040

[B33] Martin M. (2011). Cutadapt removes adapter sequences from high-throughput sequencing reads. EMBnet. J. 17, 10–12. 10.14806/ej.17.1.20028715235

[B34] McArdle B. H. Anderson M. J. (2001). Fitting multivariate models to community data: a comment on distance-based redundancy analysis. Ecology. 82, 290–297. 10.1890/0012-9658(2001)082[0290:FMMTCD]2.0.CO;2

[B35] Mehlferber E. C. Song M. J. Pelaez J. N. Jaenisch J. Coate J. E. Koskella B.. (2022). Polyploidy and microbiome associations mediate similar responses to pathogens in Arabidopsis. Curr. Biol. 32, 2719–2729.e2715. 10.1016/j.cub.2022.05.015 35640622

[B36] Müller D. B. Vogel C. Bai Y. Vorholt J. A. (2016). The plant microbiota: systems-level insights and perspectives. Annu. Rev. Genet. 50, 211–234. 10.1146/annurev-genet-120215-034952 27648643

[B37] Padukkage D. Geekiyanage S. Reparaz J. M. Bezus R. Balatti P. A. Degrassi G. (2021). Bradyrhizobium japonicum, B. elkanii and B. diazoefficiens Interact with Rice (Oryza sativa), Promote Growth and Increase Yield. Curr. Microbiol. 78, 417–428. 10.1007/s00284-020-02249-z 33083897

[B38] Price M. N. Dehal P. S. Arkin A. P. (2009). FastTree: computing large minimum evolution trees with profiles instead of a distance matrix. Mol. Biol. Evol. 26, 1641–1650. 10.1093/molbev/msp077 19377059 PMC2693737

[B39] Qi M. Berry J. C. Veley K. W. O'Connor L. Finkel O. M. Salas-González I.. (2022). Identification of beneficial and detrimental bacteria impacting sorghum responses to drought using multi-scale and multi-system microbiome comparisons. Isme J. 16, 1957–1969. 10.1038/s41396-022-01245-4 35523959 PMC9296637

[B40] Ramette A. (2007). Multivariate analyses in microbial ecology. FEMS Microbiol. Ecol. 62, 142–160. 10.1111/j.1574-6941.2007.00375.x 17892477 PMC2121141

[B41] Rong L. Y. Yao T. Feng J. Xiao-Cun D. U. Ru-Ren L. I. Cheng L. (2014). Effect of partlyreplacingchemical fertilizer by PGPR biofertilizer on pea growth. Grassland and Turf. 34, 7–12. 10.13817/j.cnki.cyycp.2014.01.002

[B42] Scheublin T. R. Sanders I. R. Keel C. van der Meer J. R. (2010). Characterisation of microbial communities colonising the hyphal surfaces of arbuscular mycorrhizal fungi. Isme J. 4, 752–763. 10.1038/ismej.2010.5 20147983

[B43] Segata N. Izard J. Waldron L. Gevers D. Miropolsky L. Huttenhower G. C. (2011). Metagenomic biomarker discovery and explanation. Genome Biology. 12, R60. 10.1186/gb-2011-12-6-r60 21702898 PMC3218848

[B44] Shekhawat K. Almeida-Trapp M. García-Ramírez G. X. Hirt H. (2022). Beat the heat: plant- and microbe-mediated strategies for crop thermotolerance. Trends Plant Sci. 27, 802–813. 10.1016/j.tplants.2022.02.008 35331665

[B45] Singha K. M. Singh B. Pandey P. (2021). Host specific endophytic microbiome diversity and associated functions in three varieties of scented black rice are dependent on growth stage. Sci. Rep. 11, 12259. 10.1038/s41598-021-91452-4 34112830 PMC8192550

[B46] Song J. Soytong K. Kanokmedhakul S. Kanokmedhakul K. Poeaim S. (2020). Antifungal activity of microbial nanoparticles derived from Chaetomium spp. against Magnaporthe oryzae causing rice blast. Plant Prot. Sci. 56, 180–190. 10.17221/41/2019-PPS

[B47] Tang J. Wu D. Li X. Wang L. Xu L. Zhang Y.. (2022). Plant immunity suppression via PHR1-RALF-FERONIA shapes the root microbiome to alleviate phosphate starvation. Embo J. 41, e109102. 10.15252/embj.2021109102 35146778 PMC8922250

[B48] Tann H. Soytong K. (2016). Effects of nanoparticles loaded with Chaetomium globosum KMITL-n0805 extracts against leaf spot of rice var. Sen pidoa. Malays. Appl. Biol. 45, 37–43.

[B49] Toju H. Peay K. G. Yamamichi M. Narisawa K. Hiruma K. Naito K.. (2018). Core microbiomes for sustainable agroecosystems. Nat. Plants. 4, 247–257. 10.1038/s41477-018-0139-4 29725101

[B50] Turrini A. Avio L. Giovannetti M. Agnolucci M. (2018). Functional complementarity of arbuscular mycorrhizal fungi and associated microbiota: the challenge of translational research. Front. Plant Sci. 9, 1407. 10.3389/fpls.2018.01407 30319670 PMC6166391

[B51] Van Deynze A. Zamora P. Delaux P. M. Heitmann C. Jayaraman D. Rajasekar S.. (2018). Nitrogen fixation in a landrace of maize is supported by a mucilage-associated diazotrophic microbiota. PLoS Biol. 16, e2006352. 10.1371/journal.pbio.2006352 30086128 PMC6080747

[B52] Wagner M. R. Tang C. Salvato F. Clouse K. M. Bartlett A. Vintila S.. (2021). Microbe-dependent heterosis in maize. Proc. Natl. Acad. Sci. U S A. 118, e2021965118. 10.1073/pnas.2021965118 34285069 PMC8325155

[B53] Walters W. A. Jin Z. Youngblut N. Wallace J. G. Sutter J. Zhang W.. (2018). Large-scale replicated field study of maize rhizosphere identifies heritable microbes. Proc. Natl. Acad. Sci. U S A. 115, 7368–7373. 10.1073/pnas.1800918115 29941552 PMC6048482

[B54] Warton D. I. Wright S. T. Wang Y. (2012). Distance-based multivariate analyses confound location and dispersion effects. Methods Ecol. Evol. 3, 89–101. 10.1111/j.2041-210X.2011.00127.x30935409

[B55] Xie Z. Zhang J. Lin Q. Liu F. Zhang C. Zhuo F.. (2019). Effect of plant growth regulators on rice lodging resistance and grain production of main-crop and ratooning rice. Chin. J. Rice Sci. 33, 158–166. 10.16819/j.1001-7216.2019.8075

[B56] Xu L. Xu J. Shao C. Wu W. Chen H. Lin W. (2020). Isolation, screening and identification of plant growth-promoting rhizobacteria from ratooning rice. J. South. Agric. 51, 814–821. 10.3969/j.issn.2095-1191.2020.04.011

[B57] Zhang J. Liu Y. X. Zhang N. Hu B. Jin T. Xu H.. (2019a). NRT1.1B is associated with root microbiota composition and nitrogen use in field-grown rice. Nat. Biotechnol. 37, 676–684. 10.1038/s41587-019-0104-4 31036930

[B58] Zhang J. Zhang N. Liu Y. X. Zhang X. Hu B. Qin Y.. (2018). Root microbiota shift in rice correlates with resident time in the field and developmental stage. Sci. China Life Sci. 61, 613–621. 10.1007/s11427-018-9284-4 29582350

[B59] Zhang T. Wang Z. Lv X. Li Y. Zhuang L. (2019b). High-throughput sequencing reveals the diversity and community structure of rhizosphere fungi of Ferula Sinkiangensis at different soil depths. Sci. Rep. 9, 6558. 10.1038/s41598-019-43110-z 31024051 PMC6484027

[B60] Zhang Z. Cai Y. Duan J. Wang Z. (2019c). Study on the Fertilizer Efficiency of Compound Biofertilizer applying in Wheat. Guangdong Agricultural Sciences.

[B61] Zhong Y. Hu J. Xia Q. Zhang S. Li X. Pan X.. (2020). Soil microbial mechanisms promoting ultrahigh rice yield. Soil Biol. Biochem. 143, 107741. 10.1016/j.soilbio.2020.107741

