# Supplementary figures and images for: Shifts in the diversity of root endophytic microorganisms across the life cycle of the ratooning rice Jiafuzhan
Source: Front Microbiol. 2023 Jun 30;14:1161263. doi: 10.3389/fmicb.2023.1161263 (PMC10348713; doi:10.3389/fmicb.2023.1161263)

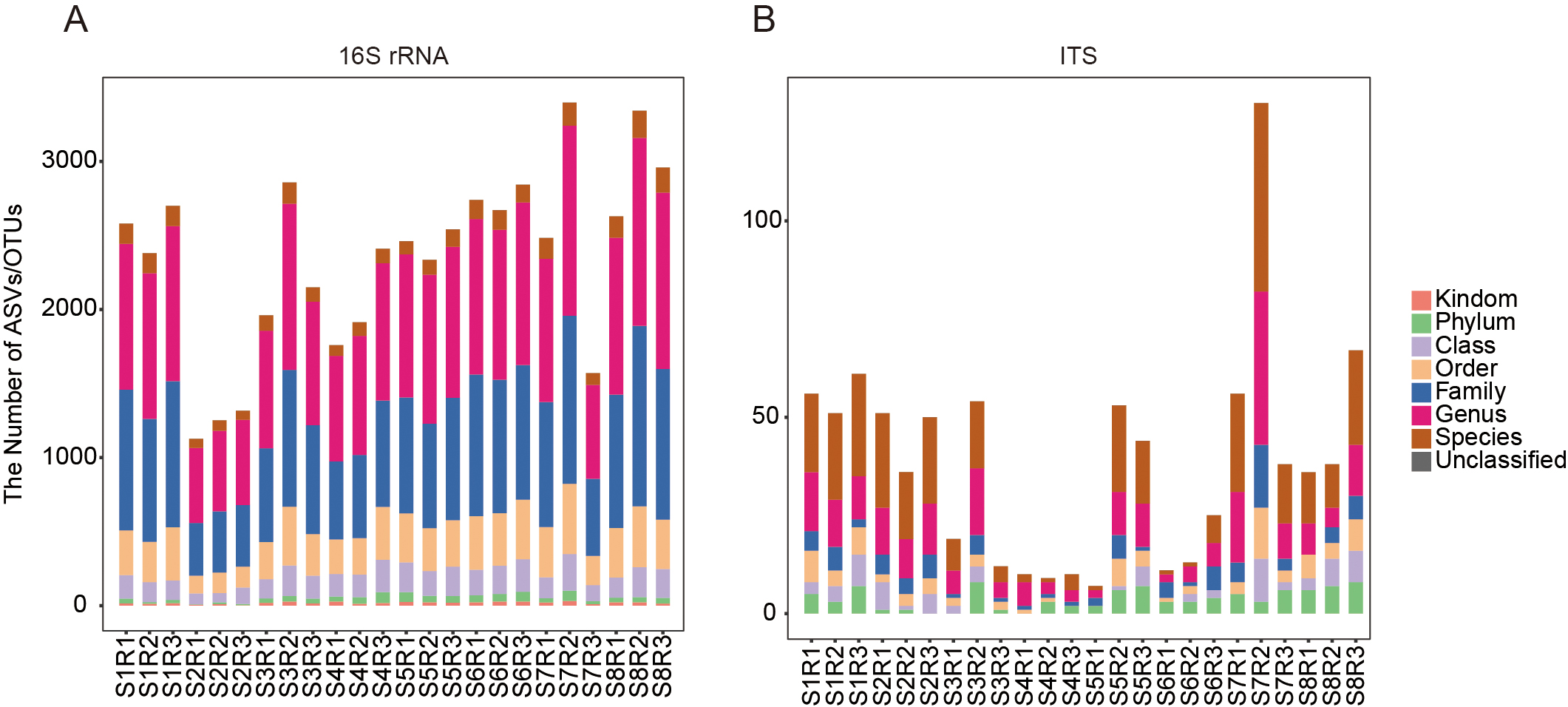

Supplement: Supplementary Figure S1 — OTU taxonomic statistics of root endophytic (A) bacteria and (B) fungi. Seedling stage samples: S1R1, S1R2, and S1R3; tillering stage samples: S2R1, S2R2, and S2R3; jointing stage samples: S3R1, S3R2, and S3R3; heading stage samples: S4R1, S4R2, and S4R3; mature stage samples: S5R1, S5R2, and S5R3; Regeneration13d samples: S6R1, S6R2, and S6R3; Regeneration25d samples: S7R1, S7R2, and S7R3; Regeneration60d samples: S8R1, S8R2, and S8R3. [file Image_1.JPEG]

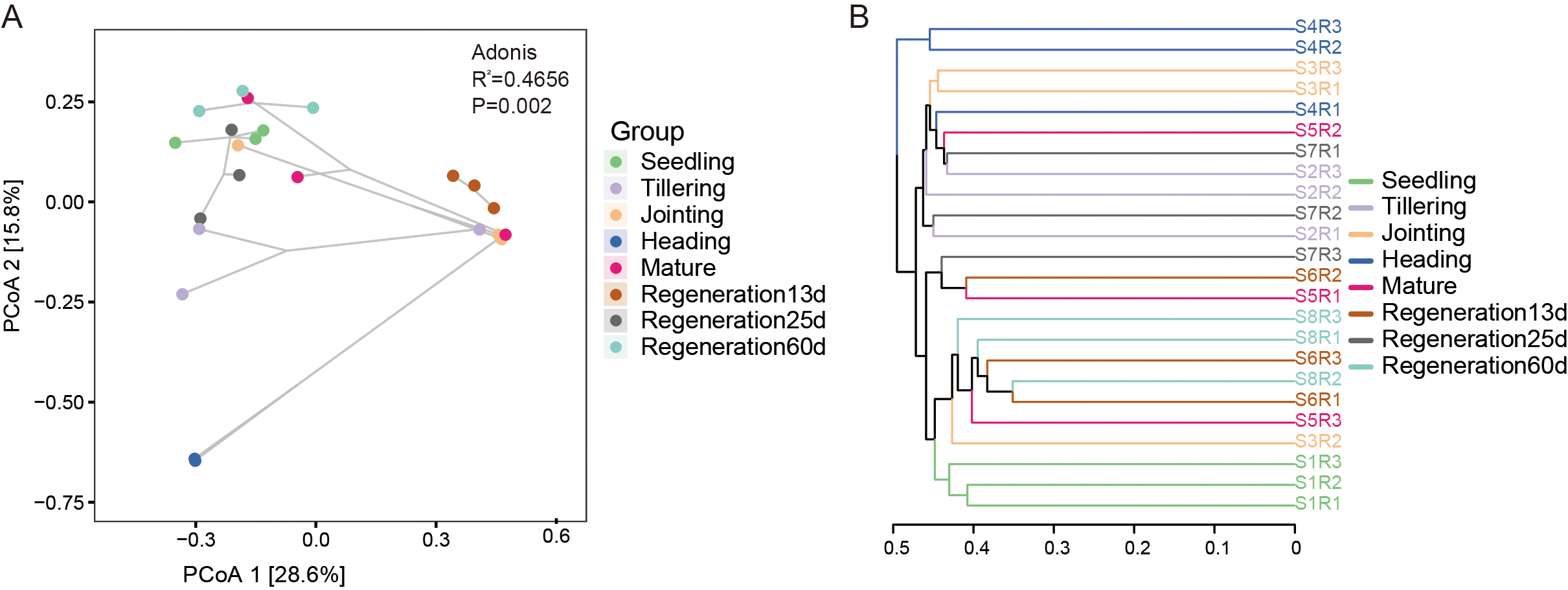

Supplement: Supplementary Figure S2 — β-diversity of root endophytic fungi at different growth stages of ratooning rice. (A) PCoA and (B) UPGMA cluster analysis. Seedling stage samples: S1R1, S1R2, and S1R3; tillering stage samples: S2R1, S2R2, and S2R3; jointing stage samples: S3R1, S3R2, and S3R3; heading stage samples: S4R1, S4R2, and S4R3; mature stage samples: S5R1, S5R2, and S5R3; Regeneration13d samples: S6R1, S6R2, and S6R3; Regeneration25d samples: S7R1, S7R2, and S7R3; Regeneration60d samples: S8R1, S8R2, and S8R3. [file Image_2.JPEG]

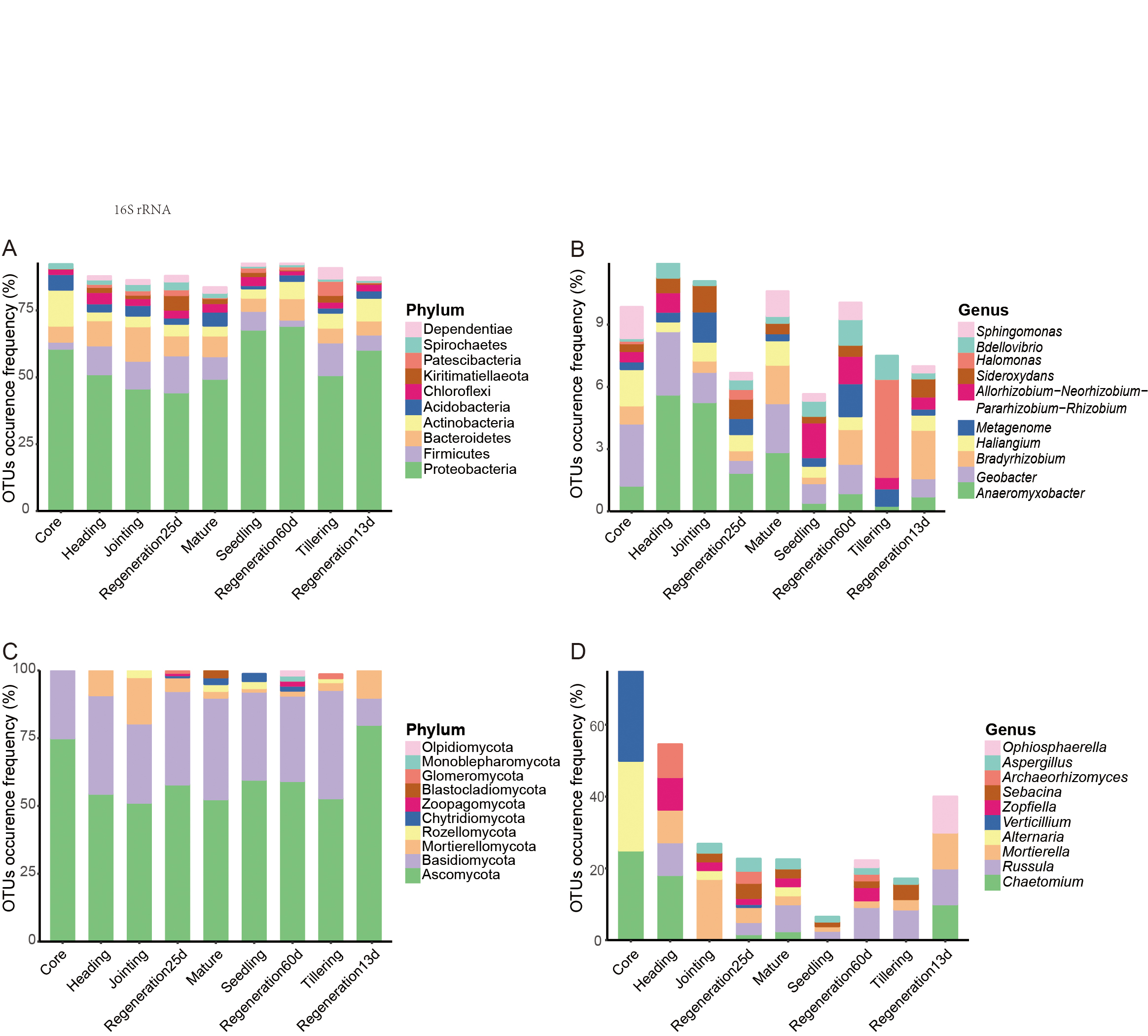

Supplement: Supplementary Figure S3 — OTU occurrence frequency of root endophytic bacterial (A) phyla and (B) genera and fungal (C) phyla and (D) genera at different growth stages of ratooning rice. [file Image_3.JPEG]

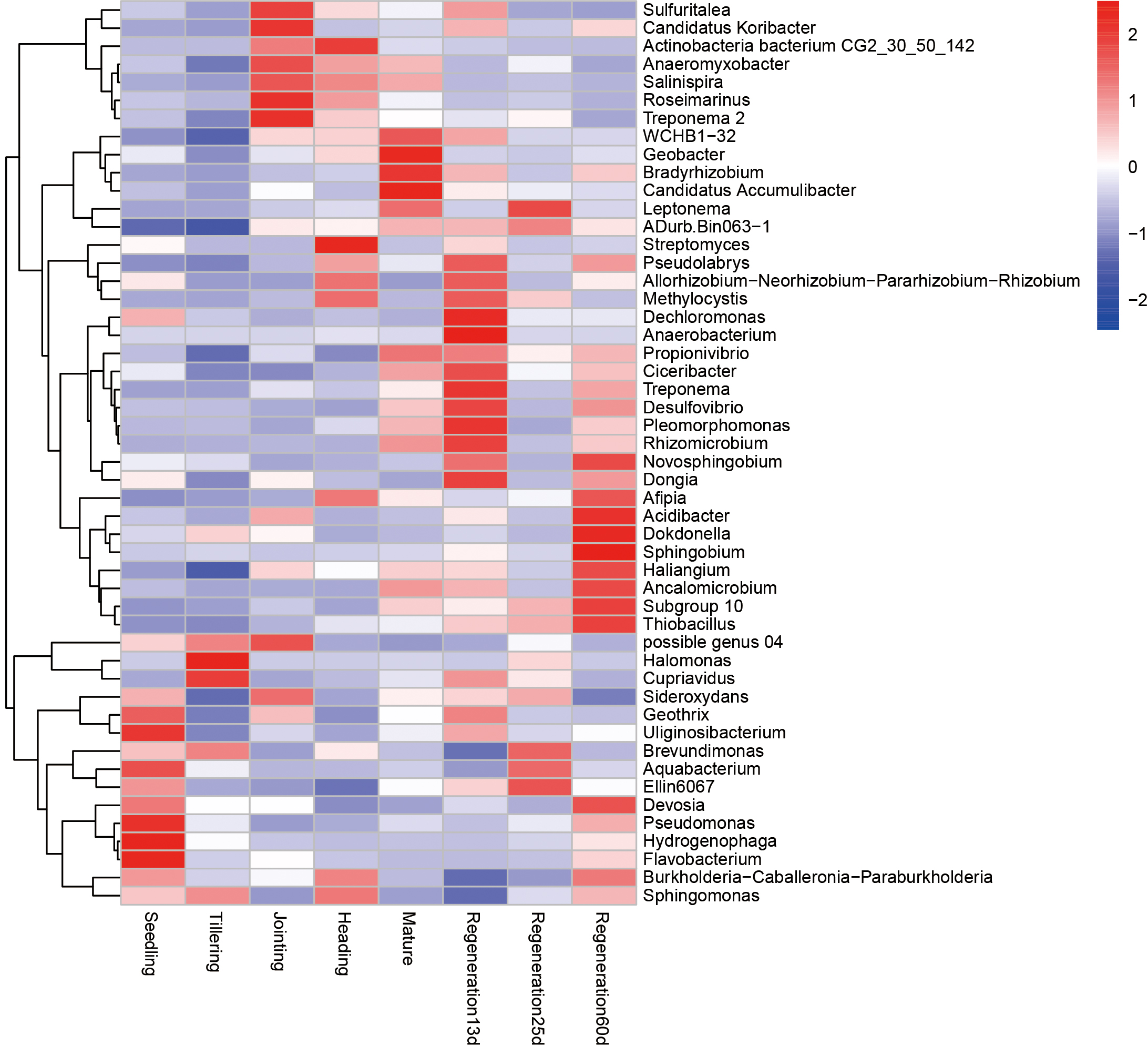

Supplement: Supplementary Figure S4 — Heatmap of the mean abundance of the top 50 root endophytic bacteria at different growth stages of ratooning rice. [file Image_4.JPEG]
